# Proctologic Surgery Prioritization After the Lockdown: Development of a Scoring System

**DOI:** 10.3389/fsurg.2021.798405

**Published:** 2022-01-26

**Authors:** Renato Pietroletti, Gaetano Gallo, Mario Muselli, Giovanbattista Martinisi, Vincenza Cofini

**Affiliations:** ^1^Surgical Coloproctology Hospital Val Vibrata, Sant'Omero (TE) and Department of Clinical and Biotechnological Sciences University of L'Aquila, L'Aquila, Italy; ^2^Department of Surgery, University of Catanzaro “Magna Grecia”, Catanzaro, Italy; ^3^Medical Statistic, Department of Life, Health and Environmental Science, University of L'Aquila, L'Aquila, Italy

**Keywords:** COVID-19, surgical priority, surgical scheduling, proctologic surgery, scoring system

## Abstract

**Introduction:**

The coronavirus disease 2019 (COVID-19) pandemic has shown a very critical impact on surgical procedures all over the world. Italy faced the deepest impact from the beginning of March 2020. Elective operations, screening, and follow-up visits had been suspended giving priority to urgent and oncologic surgery.

**Patients:**

An observational study was carried out in the Surgical Coloproctology Unit of the Val Vibrata Hospital on 152 patients awaiting a proctological surgical treatment during the national lockdown.

**Methods:**

In order to monitor the health status of patients and reschedule postlockdown surgical activities, patients were interviewed by telephone submitting a questionnaire based upon the judgment of an expert senior clinician. Following the interview, we calculated a severity index for all the proctologic diseases (hemorrhoidal disease, anal fissure, anal sepsis, slow transit or obstructed defecation, incontinence), classifying the patients according to the score. Mean age of patients was 53 (±16) years, and there were 84 males (55.3%) and 68 females (44.7%). In total, 31% of our patients suffered from anal fissure, 28% suffered from hemorrhoidal disease, 14% suffered from anal sepsis, and the remaining patients suffered from benign anorectal diseases to a lesser extent.

**Results:**

A total of 137 patients were available and divided into three classes: priority surgery (PS) with 49 patients (36.2%), deferrable surgery (DS) with 25 patients (18.1%), and long-term surgery (L-TS) with 63 patients (45.6%). There was a significant correlation between the perceived health status reported during the interview and the priority class index (Spearman's rho = 0.97, *p* < 0.001).

Differences related to age and sex were not significant (F-test = 0.43, *p* = 0.653; chi-squared test = 0.693, *p* = 0.707). 49 patients in class PS needed a prompt surgical treatment, while 24 patients allocated in class DS and 65 patients allocated in class L-TS could wait for a new ride plan for surgery.

**Conclusion:**

New tools, such as this simple score obtained during the telephone interview, can be useful for prioritization of patients on the waiting list for surgical coloproctology after the lockdown without further clinical examination and hospital access.

## Introduction

With the advent of the new coronavirus disease 2019 (COVID-19) pandemic, the strain on health systems all over the world forced to stop the majority of diagnostic procedures, elective consultations, and surgical operations (1) starting from the first week of March in Italy. Apart from emergency and oncologic procedures, nearly 90% of the elective surgical procedures canceled due to the lockdown were for benign or functional disorder (2, 3). In Italy, the waiting list for elective surgical procedures has been divided as follows into four classes based on the risk of complications or worsening of the conditions: class A to be operated within 30 days, class B to be operated within 60 days, class C to be operated within 180 days, and class D to be operated within a year. Most of elective surgical procedures for benign disease belong to classes B and C. This implies that, at the date of the lockdown, patients ready for surgery have been waiting already for 2 to 6 months.

Elective operations for coloproctological diseases suffered noticeably since they had been completely canceled at the time of the lockdown (4–6). Delayed surgery even for benign or functional disease may result in complications, unplanned emergency surgery, deterioration of individual health, disability, and social costs (7, 8).

In fact, although the prognosis is relatively good, proctological diseases such as hemorrhoidal diseases (HDs), anal fissure (AF), perianal fistula (PF), and pilonidal disease (PD) are among the most common conditions a patient may deal with.

In our surgical unit at the beginning of the lockdown, there were 152 patients on the waiting list belonging to classes B and C, affected by common colorectal and anal diseases and who had their operation canceled, waiting for the end of the emergency as well as for a reschedule of the procedure.

This study aimed to monitor the health of the patient as well as to detect possible worsening of clinical conditions needing urgent treatment through a questionnaire that all the patients filled out by telephone and which allowed us to classify the urgency of each patient. Furthermore, we aimed for the reorganization of admission of patients on a new waiting list, forecasting the end of the lockdown, and the restart of surgical activity.

## Patients and Methods

A staff doctor retrieved patients' charts from the waiting list of those 152 patients selected for surgical treatment due to common coloproctological diseases. An expert surgeon of the staff contacted the patients by telephone explaining the reason for calling and the interview started after obtaining informed verbal consent from the patient.

The first item of the questionnaire used for the telephone interview (“Would you say that in general, your health is excellent, very good, good, fair, bad, or very bad? in the last 30 days)” aimed to investigate the health status perceived by the patients (9) (Table 1). The second part of the interview was addressed to clinical information and symptoms of the coloproctological disease affecting the patient (Table 2). This part of the questionnaire was developed based on the clinical judgment expressed by the expert senior coloproctologist of the staff.

**Table 1:** Item of the telephone interview: general health.

**Identification number** **Initial** **Phone contact (home, mobile)** **Diagnosis** **Planned surgery:**		
Items		
1.	Would you say that in general your health is excellent, very good, good, fair, bad or very bad? (in the last 30 days)	Please indicate
2.	Would you say that in general your bowel is?	Normal/ diarrhea/constip
3.	If constipated, please specify daily difficult evacuation Chronic constipation abdominal distention Worsening of the above symptoms	Yes/no Yes/no Yes/no
4.	Did you see blood at defecation? If yes how often?	Yes / no Rarely/daily
5.	Did your last laboratory exams report anemia?	Yes/no
6.	Would you say that in general your perceived pain, on the numerical scale 0 to 11, with 0 being “no pain” and 11 being “the worst pain imaginable” is__?	Please indicate
7.	Do you feel something out of the anus (Prolapse)? If yes is it is…?	Yes/no Intermittent/stable
8.	Do you have anal fistula?	Yes/no
9.	Do you have a seton in place?	Yes/no
10.	Do you have discharge from the fistula? If yes, its amount is …	Yes/No Little/large
11	Do you have abscess around the fistula? (Pain, swelling, fever) If yes does this occur…	Yes/no rarely/frequently
12.	Are you taking any medication? Please specify	

**Table 2:** Construction of a severity score based on symptoms severity and frequency.

**Disease**	**Symptoms**	**Scoring**	**Priority class**
**2 A. Hemorrhoids**	Presence of prolapse	1	
	Intermittent	1	
	Stable	2	
	**Total score range**	**2–3**	**Low L-TS**
	Bleeding	2	
	Occasional	1	
	Daily	3	
	**Total score range**	**3–5**	**Deferrable DS**
	Prolapse and bleeding	3	
	Intermittent prolapse	1	
	Stable prolapse	2	
	Occasional bleeding	1	
	Daily bleeding	2	
	**Total score range**	**5–7**	**High PS**
**2 B. Fissure**	NSR of Pain 0–3	2	
	Occasional bleeding	1	
	Daily bleeding	2	
	**Total score range**	**3–4**	**Low L-TS**
	NSR of Pain 4–7	4	
	Occasional bleeding	1	
	Daily bleeding	2	
	**Total score range**	**5–6**	**Deferrable DS**
	NSR of Pain ≥8	6	
	Occasional bleeding	1	
	Daily bleeding	2	
	**Total score range**	**7–8**	**High PS**
**2C. Anal sepsis**	Seton	0	
	**Total score range**	**0**	**Low L-TS**
	Stable fistula	1	
	Low output	1	
	High output	2	
	**Total score range**	**1–3**	**Deferrable DS**
	Instable fistula	2	
	Occasional abscess	1	
	Frequent abscess	2	
	Low output	1	
	High output	2	
	**Total score range**	**4–6**	**High PS**

The answers given in the second part of the questionnaire were compared with data recorded on the chart of the patient, obtaining information concerning the evolution or stability of the disease.

Thus, as for HD, scored symptoms were prolapse, bleeding or association of both. Frequency was scored as well as occasional or frequent bleeding, intermittent, or stable prolapse.

AF was graduated with respect to pain intensity using the Numeric Rating Scale-11 (NRS-11), i.e., an 11-point scale for patient self-reporting of pain (10). The presence of bleeding, occasional, or frequent was added.

PFs were graded according to the occurrence of relapsing acute abscess and the amount and frequency of discharge.

Obstructed defecation was classified according to the Cleveland Clinic Constipation Score (CCCS) scale (11), giving priority to patients reporting higher scores at clinical evaluation (18-30) and the presence of rectal internal prolapse at anoscopy and defecography. Other conditions such as condylomata or anal stricture were considered of intermediate priority and treated accordingly at short or midterm. Pilonidal sinuses and fistulas drained with seton were deferred, unless complicated by acute sepsis or severe discharge.

The sum of the scores (priority class index) gave origin to a stratification in three classes of priority as follows: priority surgery (PS), deferrable surgery (DS), and long-term surgery (L-TS). Details of the scores for each disease are given in Table 2.

Descriptive statistics were calculated for all the variables in this study. Mean and SD were reported for continuous variables, while frequencies and percentages were reported for categorical variables. To compare sex among the three classes of priority, the chi-squared test was used, whereas the F-test for the one-way ANOVA model analysis was used to compare age. The Spearman's rho coefficient was performed to analyze the correlation between self-perceived health investigated with the first item of the telephone interview and the priority class index (PS, DS, and L-TS).

All the statistical analyses were performed using StataCorp. 2015, Stata Statistical Software: Release 14 College Station, TX, USA: StataCorp LP., setting alpha to 0.05.

## Results

**Figure 1:**
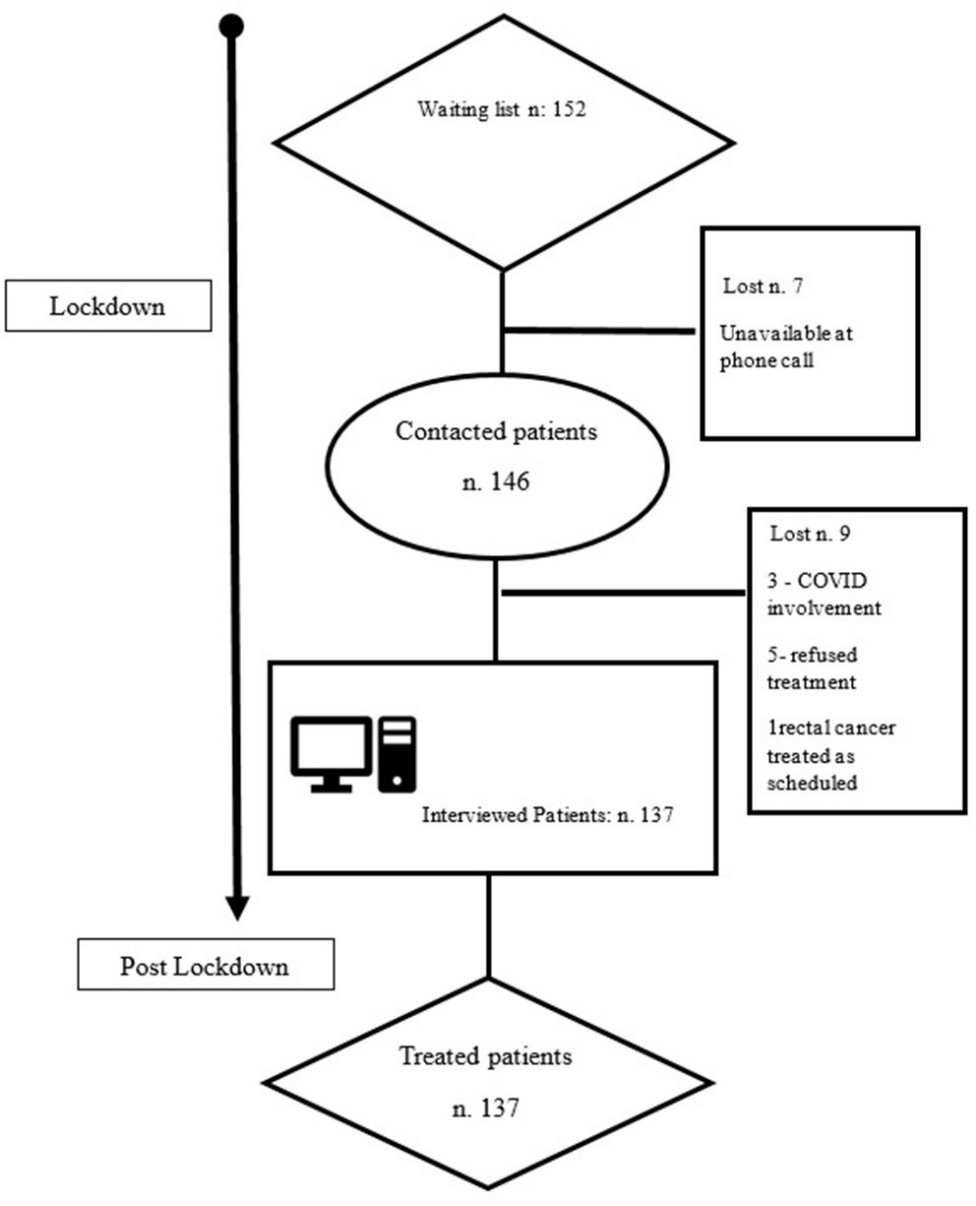
Recruitment process flowchart.

Out of 152 patients awaiting treatment in the Surgical Coloproctology Unit in the Hospital Val Vibrata, 137 patients were finally available, answered the questionnaire, and were evaluated (Figure 1).

The mean age of patients was 53 years ± 16 (SD) and there were 84 males (55.3%) and 68 females (44.7%).

Before the lockdown, the main types of diseases observed were as follows: 41 (26.9%) patients were diagnosed with III-IV degrees HDs, 45 (29.6%) with AFs resistant to medical treatment, and 23 (15.1%) with anal sepsis including one anovulvar fistula (Table 3).

**Table 3:** Data from 152 clinical records pre-lockdown (84 M, 68 F).

**Diagnosis**	***n***	**%**
III, IV degree hemorrhoidal disease	41	26.9
Anal fissure	45	29.6
Anal sepsis	23	15.1
Rectal prolapse internal/complete	5	3.2
Slow transit constipation, acquired megacolon	4	2.6
Pilonidal sinus	13	8.5
Condiloma	7	4.6
Fecal incontinence	7	4.6
Anal and rectal stricture	6	3.9
Rectal carcinoma	1	0.6
Total	152	

These data were consistent with the reported incidence of proctological and colorectal diseases at the end of the lockdown (June 2020) (Table 4). As far as self-perceived health status, most of the patients declared a very good/good health status (49.2%—fair in 11.5%). However, as many as 54 patients reported their health as bad or very bad (39.1%) (Table 4).

**Table 4:** Clinical features of patients available at interview (*n* = 137).

	***n* (%)**
Sex
Male	74 (54.1%)
Female	63 (45.9%)
Mean age (range)	51 (21–78)
Diagnosis
Hemorrhoidal disease	39 (28.4%)
Anal fissure	42 (30.6%)
Anal sepsis	20 (14.5%)
Rectal prolapse, obstructed defecation	5 (3.6%)
Slow transit constipation	3 (2.1%)
Sinus	12 (8.7%)
Condiloma	5 (3.6%)
Fecal incontinence	6 (4.3%)
Anal/rectal stricture	5 (3.6%)
Total	137
Self-perceived health
Very good/good	68 (49.6%)
Fair	15 (10.9%)
Bad/very bad	54 (39.4%)

As given in Table 5, 49 patients were classified as PS (35.7%), 25 patients were classified as DS (18.1%), while 63 patients were classified as L-TS (45.6%).

**Table 5:** Re-scheduling surgery according to the Priority Class in 137 patients.

**Diagnosis**	**Priority surgery (PS)**	**Deferrable surgery (DS)**	**Long-term surgery (L-TS)**
Hemorrhoidal disease	14 (28.5%)	7 (30.4%)	18 (27%)
Anal fissure	11 (22.4%)	13 (56.5%)	18 (27%)
Anal fistula/abscess	11 (22.4%)	3 (28.6%)	7 (10.7%)
Rectal prolapse/Obstructed defecation	2 (4%)	1 (4.3%)	2 (3%)
Severe constipation/adult megacolon	3 (6.1%)	0 (0%)	1 (2%)
Pilonidal Sinus	0 (0%)	0 (0%)	12 (18.4%)
Condilomata	5 (10.2%)	0 (0%)	0 (0%)
Incontinence and Ectropion	2 (4%)	0 (0%)	4 (6.1%)
Anal stricture	1 (2%)	0 (0%)	4 (6.1%)
Total 137	49 (35.7%)	23 (16.7%)	65 (47.4%)

The mean age of patients classified in PS category was 53 (±15) years and they were older than patients classified as DS and L-TS, respectively (52 ± 11) and (50 ± 17); the differences were not significant (F-test = 0.43; *p* = 0.653).

The distribution between males and females was not different by classes (Pearson's chi-squared test = 0.6926; *p* = 0.707) and there was a high positive correlation between the self-perceived health and the estimated priority index (Spearman's rho = 0.97, *p* < 0.001).

Patients affected by HD in class PS complained mainly of daily and severe bleeding, leading to anemia in four patients, whereas those affected by AF reported high scores (8–11) at pain evaluation with daily, frequent analgesic intake (2.8 daily doses + −0.5; range 0–4).

As for obstructed defecation, the CCCS score of 25 gave priority for surgical treatment in one patient with internal rectal prolapse [stapled transanal rectal resection (STARR)]. Another female patient affected by complete rectal prolapse had a Delorme procedure. In severe chronic constipation with dolichomegacolon, three out of four patients complained of recurrent episodes of acute intestinal subocclusion. In these patients, the diameter of the ascending colon was larger than 10 cm on plain, abdominal X-rays and, thus, surgical treatment was planned in class PS (subtotal colectomy and ileorectal anastomosis) after a short course of medical treatment for two patients.

The patients within class PS were treated consecutively at reopening of elective surgery starting from the first week of June. 11 cases out of 50 cases showed a progression of the disease compared to the prelockdown evaluation and this made a change in surgical treatment as follows: three patients waiting for dearterialization moved to excisional hemorrhoidectomy, four fistula patients planned for ligation of intersphincteric fistula tract necessitated drainage of recurrent acute sepsis and seton placement instead, and three patients with AF were given anoplasty in place of lateral sphincterotomy due to deepening of the fissure and enlargement of skin tag and sentinel polyp.

In those cases belonging to the DS category and operated between August and September, we noticed an evolution of the disease although to a lesser extent; three patients affected by AF showed local sepsis and were treated by means of drainage, posterior sphincterotomy, and anoplasty. Interestingly, none of those three patients complained of a substantial change of symptoms and, thus, of the score.

Anoplasty was performed in three patients affected by anal stricture (one in the PS group and two in the DS group) or in the case of mucosal ectropion (2 patients in the DS group). In two of them, incontinence was associated and treated by sphincteroplasty or sphinkeeper placement.

Finally, five patients affected by large anoperineal condylomata were treated by means of diathermic excision. The patient affected by low rectal cancer was treated at the end of neoadjuvant treatment as planned since oncologic surgery was not affected by the lockdown and underwent an abdominoperineal amputation.

In the L-TS category, all the operations were performed as planned previously when patients were selected for operation. In this respect, medical treatment or conservative procedures helped notably as a bridge to surgery.

## Discussion

The occurrence of the COVID-19 pandemic implied partial or complete cancelation of diagnostic procedures, outpatient visits, and elective surgical operations starting from the beginning of March 2020. Elective surgery for benign conditions faced a cancelation of procedures to an estimate of 71.2–87.4% (2). The practice of surgical coloproctology was heavily affected by this lockdown, since most operations performed in this area were for benign, functional disorders (4–6).

At the beginning of the lockdown, patients placed on the waiting list in our institution belonged to classes B or C according to our “national plan for management of waiting lists” (12). We aimed to establish criteria for reclassifying the priority of patients in view of the end of the lockdown. In addition, we had the opportunity to monitor the clinical conditions of patients, acting promptly for urgent treatment in case of complications or progression of the disease.

At the end of the lockdown, June in our area, patients who had their operation canceled deserved a prompt evaluation to reassess their conditions in view of the time passed. To make more than a hundred visits to re-evaluate and reschedule patients in a new order of priority, also in consideration of the restrictions imposed by the COVID-19 pandemic still going on, it would have been challenging or even impossible.

Our scoring system helped in reassessing the conditions of the patient by means of a simple telephone call, also comparing the answers with patient's chart handled by us and containing relevant clinical data. We observed concordance between the severity of disease and a bad health status declared by the patient.

Self-perception of health status in a patient judged as bad, fair, or good is a trustable predictor of reduced functional capacity, depression, increased search for hospital care, and even mortality (9).

Perceived health is an overall indicator of the general health status of a population. Our results are in line with what is reported in the literature. In fact, several studies have highlighted the association of the perception of the state of health, with mortality, morbidity, functional decline, and higher request of health services resources (13–15).

In total, 39% of patients felt their health status was bad. This result is difficult to understand since the mean age of patients was not very old. However, in this respect, stress played a role due to the lockdown itself and to the COVID-19 pandemic.

Giani et al. divided patients into deferrable and not deferrable based on both the symptoms and COVID-19 infection risk. Interestingly, 198 out of 548 (36.1%) visits were canceled, and there was a 55% increase of office-based procedures (29 visits in 2019 and 45 visits in 2020).

None of the patients or surgeons resulted COVID-19 positive (16).

With the adoption of the scoring system, we obtained a new stratification in three different classes of patients in each disease requiring different priorities. Indeed, benign proctological diseases such as HD, AF, PF, and PD covered 73% of patients. Our simple score helped in identifying those patients in each group necessitating operation as soon as activity was resumed.

The results of our interview showed that, in the sample of 138 patients contacted after the lockdown, there were 50 patients in class PS ready for operations, while 63 patients were in class L-TS could wait for a new ride plan to access the hospital care.

With the restart of elective surgery, the operations were performed accordingly to the new priority order in June and July whereas, in August and September, patients in DS underwent surgical treatment.

As expected, we observed in class PS a certain degree of progression and worsening of clinical conditions of patients, affecting 13% of the total of patients with HD, AF, and PF and leading to a change in surgical strategy. In class DS, we found a progression of the disease and worse clinical conditions only in a minority of cases as compared to the prelockdown selection.

Indications for surgery in case of benign surgical disorders are usually posed after a failed course of conservative measures; in this respect, the availability of alternative, nonoperative treatments or tools is to be considered, since they may represent a tamponade treatment waiting for surgery (17).

With the restrictions imposed in hospital access by the COVID-19 pandemic and the subsequent lockdown, our telephone interview helped in monitoring the health of patients and it was extremely useful in prioritizing again patients after initial evaluation. The questions of the interview are quite basic; therefore, it can be conducted by trained, nonspecialist personnel. In this respect, straightforward monitoring of the conditions of the patient, especially for those in a long-standing surgical waiting list, can be adopted independently from the present situation. This may be helpful to prevent worsening of the diseases that, if occurring, shift the treatment to emergency with a rise in complications and costs.

Our results were consistent with the current growth trend manifested toward telemedicine, which has helped to overcome the burden of the COVID-19 pandemic (18–20).

This study has some limitations; the main one is represented by the need for a strong statistical validation of the simple score proposed. However, at the end of the lockdown, there was a strong need for reprogramming surgical activity, and our score showed to be a useful and simple method for this goal. In addition, the small size of subjects selected in a subspecialty unit may be a limit, although they represent a rather homogeneous group of patients. The easy and friendly use of our interview showed to be effective in prioritization a good amount of elective surgical operations minimizing the negative effect of the lockdown. Adoption of measures such as priority scores may enable to maintain a certain volume of elective surgery, despite restrictions due to the COVID-19 pandemic.

In conclusion, recovery plans for elective surgery after the lockdown are to be fundamentally prepared in all the areas of routine surgery for benign diseases. This is of the utmost importance also considering possible subsequent relapses of COVID-19 disease leading to repeated lockdown and cancelation of planned surgery. In replanning elective surgery during the COVID-19 pandemic, resources limitations and risk of COVID-19 transmission must be considered as additional adverse factors in limiting hospital access and surgical treatment.

## Data Availability Statement

The raw data supporting the conclusions of this article will be made available by the authors, without undue reservation.

## Author Contributions

All authors listed have made a substantial, direct, and intellectual contribution to the work and approved it for publication.

## Conflict of Interest

The authors declare that the research was conducted in the absence of any commercial or financial relationships that could be construed as a potential conflict of interest.

## Publisher's Note

All claims expressed in this article are solely those of the authors and do not necessarily represent those of their affiliated organizations, or those of the publisher, the editors and the reviewers. Any product that may be evaluated in this article, or claim that may be made by its manufacturer, is not guaranteed or endorsed by the publisher.
